# Mitochondrial diversity of Bulgarian native dogs suggests dual phylogenetic origin

**DOI:** 10.7717/peerj.5060

**Published:** 2018-06-27

**Authors:** Miroslav Marinov, Denitsa Teofanova, Dimitar Gadjev, Georgi Radoslavov, Peter Hristov

**Affiliations:** 1 Institute of Biodiversity and Ecosystem Research, Bulgarian Academy of Sciences, Sofia, Bulgaria; 2 Department of Biochemistry, Faculty of Biology, Sofia University “St. Kliment Ohridski”, Sofia, Bulgaria; 3 Agricultural and Stockbreeding Experimental Station, Agricultural Academy, Smolyan, Bulgaria

**Keywords:** Bulgarian native dog, D-loop region, Genetic diversity, Population structure

## Abstract

The dog has been the first domesticated animal to have a central role in human society from ancient times to present day. Although there have been numerous investigations of dog phylogeny and origin, genetic data of dogs in the region of the Balkan Peninsula (South-Eastern Europe) are still scarce. Therefore, the aim of the present study was to perform phylogenetic analysis of three native Bulgarian dog breeds. A total of 130 samples were analyzed at HVR1 (hypervariable region, D-loop region). The samples were taken from two hunting dog breeds (Bulgarian Hound Dog: Barak, *n* = 34; Bulgarian Scenthound Dog: Gonche, *n* = 45) as well as from a Bulgarian Shepherd Dog (*n* = 51). The first two breeds are reared in a flat region of the country (the Northern part of Bulgaria, the Danubian Plain), while the last breed is a typical representative of the mountainous part of the country. The results have shown the presence of almost all main clades—A, B, C and D—in the three dog breeds taken together, except clades E and F, as expected. With regard to haplogroups distribution, there are clear differences among investigated breeds. While hunting breeds exhibit a prevalence of clade C, the mountainous Shepherd dog shows presence of the D2 haplogroup but absence of the C clade. In conclusion, the present study has been the first to investigate the mitochondrial DNA diversity of native dog breeds in Bulgaria. The results have revealed a clear difference of haplogroups dissemination in native hunting and shepherd dogs, which suggests a dual independent phylogenetic origin, without hybridization events between these dogs.

## Introduction

The origin and evolution of the domestic dog has remained for a long time a controversial question for the scientific community with regard to basic aspects such as the place of origin (Wang et al., 2016). It is known that two main processes have occurred in dog (*Canis lupus familiaris*) history: first, primitive dogs were domesticated from their wild predecessor, the gray wolves (*Canis lupus lupus*) before the beginning of the last ice age (about 30,000 BP). Second, the primitive forms were further selected to form many dog breeds with specialized abilities and morphology at the beginning of the Early Neolithic period (about 10,000 BP) (Lindblad-Toh et al., 2005; Wang et al., 2014; Wang et al., 2016; Wayne & Ostrander, 2007). Despite the numerous efforts to study dog phylogeny and evolution, a basic question that still needs to be elucidated is related to the origin and evolution of the domestic dog. In this aspect, several different geographical regions have been proposed as the main domestication centers.

The first such investigations of the geographical origin of dogs were based on maternally transmitted mitochondrial DNA (mtDNA) in modern dogs, which indicated that dogs originated in the southern part of East Asia (Savolainen et al., 2002; Pang et al., 2009). However, several subsequent studies based on mtDNA from ancient dog samples have suggested Europe as the place of origin (Thalmann et al., 2013). Using genome-wide genotyping of modern dogs, the Middle East has been proposed as the main center of dog domestication, in contrast to findings from using mtDNA sequence data (VonHoldt et al., 2010).

The second process after dog domestication was selection and creation of new breeds, specialized for the different requirements of new human societies. During the Early Neolithic hunter-gatherer human lifestyle was replaced by the emergence of early farmers. For Europe, this process started about 8,000 BP in South-Eastern Europe (the Balkans) as the closest point to Anatolian settlements (Mathieson et al., 2018). The contemporary view of ancient human population in South-Eastern Europe (during Paleolithic, Mesolithic, Neolithic, Copper, Bronze and Iron Age) is that the Balkan human population is similar to the Anatolian population, but different from those in the North Pontic Steppe and West Europe (Mathieson et al., 2018). During this period, and later in the antique period, there was migration of human populations from Eastern Europe to the South direction (the Balkans). The migration process is quite possibly connected with dog dissemination and breeding. Consequently, due to this migration, there was mixing between South-European and Anatolian, on the one hand and Middle European and on the other hand human and dog populations (Frantz et al., 2016; Botigué et al., 2017). MtDNA analysis of the domestic dog has revealed six main clades, named A, B, C, D, E and F (Savolainen et al., 2002). Clades A, B, and C contain 95.9% of all dog haplotypes and are represented worldwide (clade A), or everywhere except America (clades B and C) (Pires et al., 2006). Clades E and F have a limited geographic distribution (East Asia) with low frequencies (Savolainen et al., 2002). Clade D consists of two subclades, D1 and D2, which have a limited geographic distribution, specific to Europe and the Middle East. Subclade D1 is restricted to North Europe (Scandinavia), while sublade D2 is found in the Mediterranean region and the Middle East (Savolainen et al., 2002), and in the North Africa (Adeola et al., 2017) The subclades of A (A1–A6), B (B1 and B2) and C (C1 and C2) also have specific geographic distribution in Eurasia. For example, subclades A2a, A2b, A3, A4, A5, B2a and C1b have an East Asian distribution, while A1a1, A1b1, A1b2, B1a1a, B1a1, C1a2, C1a4, C2a2, C2a3 are specific to Europe (Duleba et al., 2015).There are three Bulgarian native dog breeds—the Bulgarian Shepherd Dog (BShD), the Bulgarian Barak Hound (BBH, Barak) and the Bulgarian Scenthound Dog (BSD, Gonche).

The hunting dogs are some of the oldest hunting dogs in the Balkans. Presumably, they originated back in the Thracian period (around 2500 BC), based on pictures from this epoch. In general, on the territory of the Balkan Peninsula, hounds are divided into smooth- and rough-haired.

In Bulgaria, one of the most widely distributed smooth-haired hunting dog breeds is the Bulgarian Scenthound dog (BSD, Gonche). The breed is also known as “Bicolor hound” or “Ludogorsko gonche” due to the area where it is largest populations are found—in the Ludogorie region in Northeastern Bulgaria. Dogs were used for hunting of large and small hairy game and predators. Some of the nearest members of this breed are the Hungarian hound (Transylvanian Scenthound), the Serbian Hound (Tricolor hound) and the Greek Harehound.

The Barak (Bulgarian Barak Hound, BBH, Barak) is a rough-haired Bulgarian dog. The Barak belongs to the group of hounds chasing game over a freshly traced trail. This breed is prevalent in the Central, North and North-Western (the Danubian Plain) parts of the country. Some most closely related member of this breed is the Slovak Rough-haired Pointer.

The Bulgarian Shepherd Dog (BShD) is a traditional mountain livestock guard dog breed, usually named Karakachan Shepherd. Other names are Ovcharsko Kuche and Thracian Mollos. It is possible that the Balkan shepherd dogs had a direct predecessor, known as the now extinct Molossus dog (Kitchell Jr & Kenneth, 2014). The Molossus originated on the Balkans as described by many old authors as Schöps (1937) who wrote about it in the German cynological magazine “Zeitschrift für Hundeforschung”. The nearest members of this breed are the Tornjak (Bosnia and Herzegovina and Croatia), the Sharplaninac (FR Macedonia), and the Akbash Dog (Turkey).

A newly created dog breed based on the Karakachan Dog (BShD) is the Bulgarian Shepherd Dog (BOK). Other breeds, such as the Central Asian Shepherd Dog (Alabai), the Caucasian Shepherd dog, the St. Bernhardshund, the Newfoundland, etc., have also taken part in the development of the breed. Bulgarian Shepherd Dog was created mainly as a very large companion dog, suitable for urban environment and the exhibition ring. There is no evidence that it was used as a traditional shepherd dog.

The aim of this study is to reveal mtDNA variation in three Bulgarian native dog breeds with respect to their phylogenetic origin. The obtained genetic profile and distribution of the main subclades among these breeds are compared with other related geographic populations.

## Materials and Methods

### Population structure and sample collection

**Figure 1:**
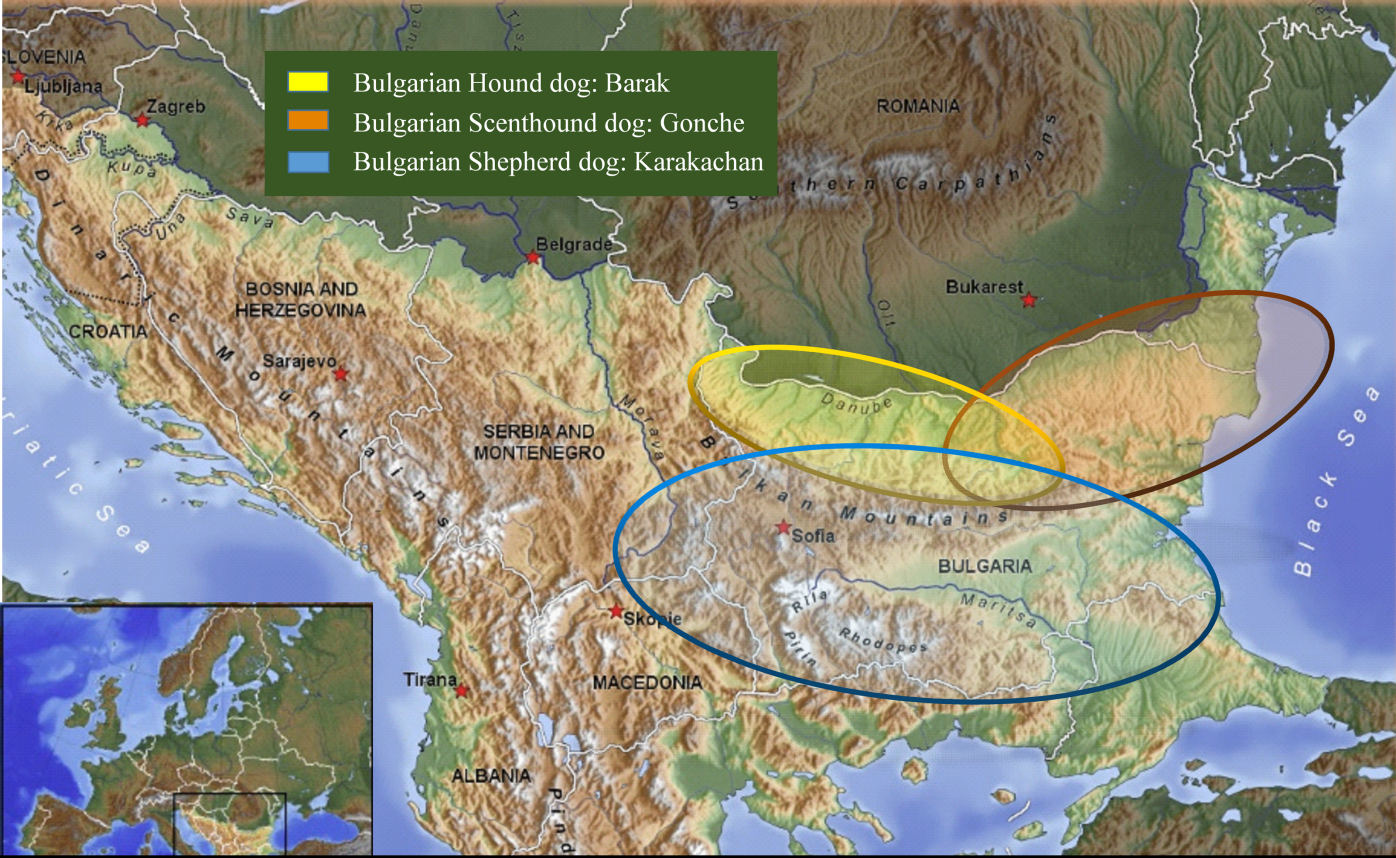
Map showing samples location.

The experiments had been carried out under permissions and the guidelines of the Bulgarian Academy of Sciences and the Bulgarian Ministry of Environment and Waters (no. 627/30.03.2015). Hair from 130 animals belonging to four different breeds or populations of dogs were collected from breeding kennels, and from distinct geographic locations in their historical breed regions (Table 1, Fig. 1). The hair samples from the new breed of the **Bulgarian Shepherd Dog** (**BOK**, *n* = 14) were taken from three kennels under the control of the Bulgarian Republican Federation of Cynology: “Zapryan”—Vasil Gajdarov, “Sredets”—Tihomir Blagoev, and “Aviyul”—Avram Petkov. The hair samples of the other three breeds were taken as follows: the **Bulgarian Shepherd Dog** (**BShD**, *n* = 37)—from “Goran”, Dimitar Draganov; the **Bulgarian Barak Hound** (BBH, **Barak**, *n* = 34)—“Kan el Kot”, Hristo Kanev, “Vom Pirin Hunt”, Asen Belezhkov; National club, **Bulgarian Scenthound dog** (BSD, **Gonche**, *n* = 45)—“Zhelezni”, Dimitar Dimitrov, “Kaimakanski”, Slavi Kajmakanski.

**Table 1:** Information on the dog breeds used in this study. GD, guarding dogs; LHD, livestock herding dogs; HD, hunting dogs. Demographic data only available for Bulgarian dog breeds.

Breed/population of dogs	Number of samples	Locality	Function	Current female population size	Current conservation status	Total number of haplotypes^a^	Unique haplotypes^b^
Bulgarian Shepherd dog: BOK	14	All country	GD	1,300	Not at risk	11	4
Bulgarian Shepherd dog: Karakachan	37	The Rhodope mountain and other mountainous parts of the country	LHD	100	Endangered	10	5
Bulgarian Hound dog: Barak	34	North-Western Bulgaria	HD	120	Endangered	15	7
Bulgarian Scenthound dog: Gonche	45	North-Eastern Bulgaria	HD	150	Endangered	10	3

**Notes.**

a Based on 732 bp mtDNA fragment calculated by DnaSP 5.10.1 (Librado & Rozas, 2009).

b Unique haplotypes based on the clipped 630 bp mtDNA fragment described above.

Animals were selected based on morphological standards and on information about their pedigree in order to exclude related animals. All dog lineages originate from different regions of the country, which is confirmed by kennels and dog associations.

### DNA extraction, PCR amplification and sequencing

Total DNA was isolated from hair follicles by using a GeneMATRIX Tissue and Bacterial DNA purification Kit (Cat. No. E3551-01; EURx Ltd., Gdansk, Poland) according to the manufacturer’s instructions. Briefly, hair was cut into pieces (up to 100 roots). After that, the hair was digested in a lysis buffer (a component of the DNA purification kit), 20 µL of 1M DTT and proteinase K, and incubated overnight at 56 °C. The extracted DNA was resuspended in 50 µL of an elution buffer. The DNA concentration was determined spectrophotometrically, and the quality of the DNA samples was examined on 1% agarose gel electrophoresis stained with Greensafe premium (Cat. No. MB13201; Nzytech, Lisbon, Portugal). The purified DNA was stored at −20 °C until PCR assay.

The mtDNA D-loop region was amplified with primers: HVI-F15453 5′-CCCTGACACCCCTACATTCA-3′ and HVI-R16107 5′-CCATTGACTGAATAGCAC CTTGA-3′ designed by Vila, Maldonado & Wayne (1999). The PCR mixture contained 25 µL of NZYTaq 2× Colourless Master Mix (Cat. No. MB04002; Nzytech, Lisbon, Portugal), 0.4 µM of each primer (FOR/REV), 1 µL of template DNA in a total volume of 50 µL. All PCR reactions were carried out using a Little Genius thermocycler (BIOER Technology Co., Ltd, Hangzhou, China) under the following conditions: initial denaturation at 94 °C for 5 min; 30 cycles (denaturation at 94 °C for 30 s; primer annealing at 50 °C for 30 s; extension at 72 °C for 1 min) and final extension at 72 °C for 10 min. PCR products were visualized on a 2% agarose gel with Greensafe premium (Cat. No. MB13201; Nzytech, Lisbon, Portugal). The fragment size was determined using Gene-Ruler™ 100 bp Ladder Plus (Cat. No. SM0323; ThermoFisher Scientific Inc., Waltham, MA, USA).

The successfully amplified products were purified by a PCR purification kit (Gene Matrix, PCR clean-up kit, EURx, Poland) and sequenced in both directions by a PlateSeq kit (Eurofins Genomics, Ebersberg, Germany).

### Statistical and phylogenetic analysis

All 130 sequences were manually edited and aligned by MEGA software version 7.0 (Kumar, Stecher & Tamura, 2016) using the dog mtDNA sequence NC_002008 as a reference (Kim et al., 1998). The obtained sequences (about 730 bp in length from covered tRNA-Pro genes and the beginning of the D-loop region, HV1) included in this study were deposited in the National Center for Biotechnology Information (NCBI) GenBank database under accession numbers (NCBI: MG920357–MG920486). Sequences were analyzed by polymorphic SNPs, and haplogroups were determined according to Duleba et al. (2015) and Song et al. (2016) as well as MitoToolPy program (Peng et al., 2015), (http://www.mitotool.org/mp.html) with reference sequence EU789787 (Pang et al., 2009). The statistical quantities for the DNA sequences, including number of haplotypes and haplotype diversity, nucleotide diversity and Fu and Li’s D and F test were performed by using DnaSP 5.10.1 (Librado & Rozas, 2009). Phylogenetic relationships of mtDNA haplotypes were explored by a Reduce Median network using NETWORK 4.5.1.6 (Fluxus Technology Ltd.) (available at http://fluxusengineering.com). The evolutionary distances were computed using the Maximum Composite Likelihood method and were within the units of the number of base substitutions per site. The percentage of replicate trees in which the associated taxa clustered together in the bootstrap test (10,000 replicates) is shown next to the branches. All positions containing gaps and missing data were eliminated.

Principal component analyses (PCA) were performed using Excel software implemented by XLSTAT, as described elsewhere (Achilli et al., 2007). The PCA were carried out: one—by considering only our sample.

## Results

### Genetic diversity and differentiation of Bulgarian dogs

The primers: HVI-F15453/HVI-R16107 amplified a sequence comprising tRNA-Pro genes and the beginning of the D-loop region (HVR1) at the 5′ end of the control region (about 630 bp). All sequences covered from 15,361 to 16,092 bp according to Ref. sequence accession number NC_002008 (Kim et al., 1998). We also observed in all dog sequences an insert at the position 15,514 bp according to Ref. sequence accession number EU789787 (Pang et al., 2009). This insert covered 23 bp similar to Ref. sequence accession number NC_002008. Thirty-eight different haplotypes were obtained from all 130 individuals (accession number MG920357–MG920486) (Table 1). We identified 19 unique haplotypes in all 130 sequences (Table 1, Supplemental Information 1). The largest number of them, seven, was found in the Bulgarian Hound Dog: Barak, while the least—three unique haplotypes—were found in the Bulgarian Scenthound Dog: Gonche (Table 1, Supplemental Information 1).

The coefficient of diversity within all samples was calculated to be 0.014 ± 0.0022 (Kumar, Stecher & Tamura, 2016). The mean group distance varied from 0.019 in the Bulgarian Shepherd Dog: BOK to 0.009 in Bulgarian Shepherd Dog: Karakachan. The highest value of mean distance between populations was calculated as 0.017 between the Bulgarian Shepherd Dog: BOK, the Bulgarian Hound Dog: Barak, and the Bulgarian Scenthound Dog: Gonche. The lowest value 0.013 of the mean distance was observed between the Karakachan and the Gonche.

The Bulgarian Shepherd Dog (BOK) showed the highest value of haplotype diversity (0.934 ± 0.0037), followed by the Bulgarian Hound Dog Barak (0.916 ± 0.0006) (Table 2). Among the remaining dog breeds, none showed haplotype diversities higher than 0.90.

**Table 2:** Haplotype (H) and nucleotide (*π*_*n*_) diversity, mean number of pair-wise differences (*π*) between haplotypes within populations, polymorphic sites (p.s.), number of haplotypes (Hn), and Fu and Li’s D and F tests in four Bulgarian dog breeds.

Breed	H ± SD	*π*_*n*_	*π*	p.s.	Hn	Fu and Li’s D	Fu and Li’s F
Bulgarian Shepherd dog: BOK	0.934 ± 0.0037	0.0190	13.835	55	11	−1.461	−1.495
Bulgarian Shepherd dog: Karakachan	0.812 ± 0.0016	0.0087	6.366	26	10	1.191	0.967
Bulgarian Scenthound dog: Gonche	0.821 ± 0.044	0.0173	5.459	15	10	1.591	1.865
Bulgarian Hound dog: Barak	0.916 ± 0.0006	0.0137	10.029	43	15	−1.146	−0.999

Nucleotide diversity per site (*π*_*n*_) was on average high, ranging from 0.019 (Bulgarian Shepherd Dog: BOK) to 0.0087 (Bulgarian Shepherd Dog: Karakachan). According to Vila, Maldonado & Wayne (1999), this statistic is not highly sensitive to sample size. The mean number of pairwise differences (*π*) between haplotypes within populations varied from 13.83 (Bulgarian Shepherd Dog: BOK) to 5.45 (Bulgarian Scenthound Dog: Gonche). The highest number of haplotypes (Hn), 15, was observed in the Bulgarian Hound Dog: Barak, and the lowest, 10, in the Bulgarian Shepherd Dog: Karakachan and the Bulgarian Scenthound Dog: Gonche.

The Fu and Li’s FL-D and FL-F tests showed the lowest values in the BOK dog, which was not surprising, because other dog breeds were included in the creation of that breed (Table 2).

We defined 38 haplotypes in all dog breeds, 19 of which were unique (Table 2). All unique haplotypes were represented with only one sample, while haplotype H36 and H37 were represented with four samples belonging to subhaplogroup D2b (Supplemental Information 1).

In order to graphically display (and summarize) the mitochondrial relationships among the analyzed breeds, we performed a principal component analysis (PCA).

The PCA plot clearly separated the two investigated dog breeds: hunting and shepherd (Supplemental Information 1). The hunting dogs: Gonce and Barak are grouped closely, while the BOK and Karakachan dogs are rather distant. The PCA also supported the hypothesis of the independant origin of the investigated hunting and shepherd dog breeds.

### Phylogenetic analysis

**Figure 2:**
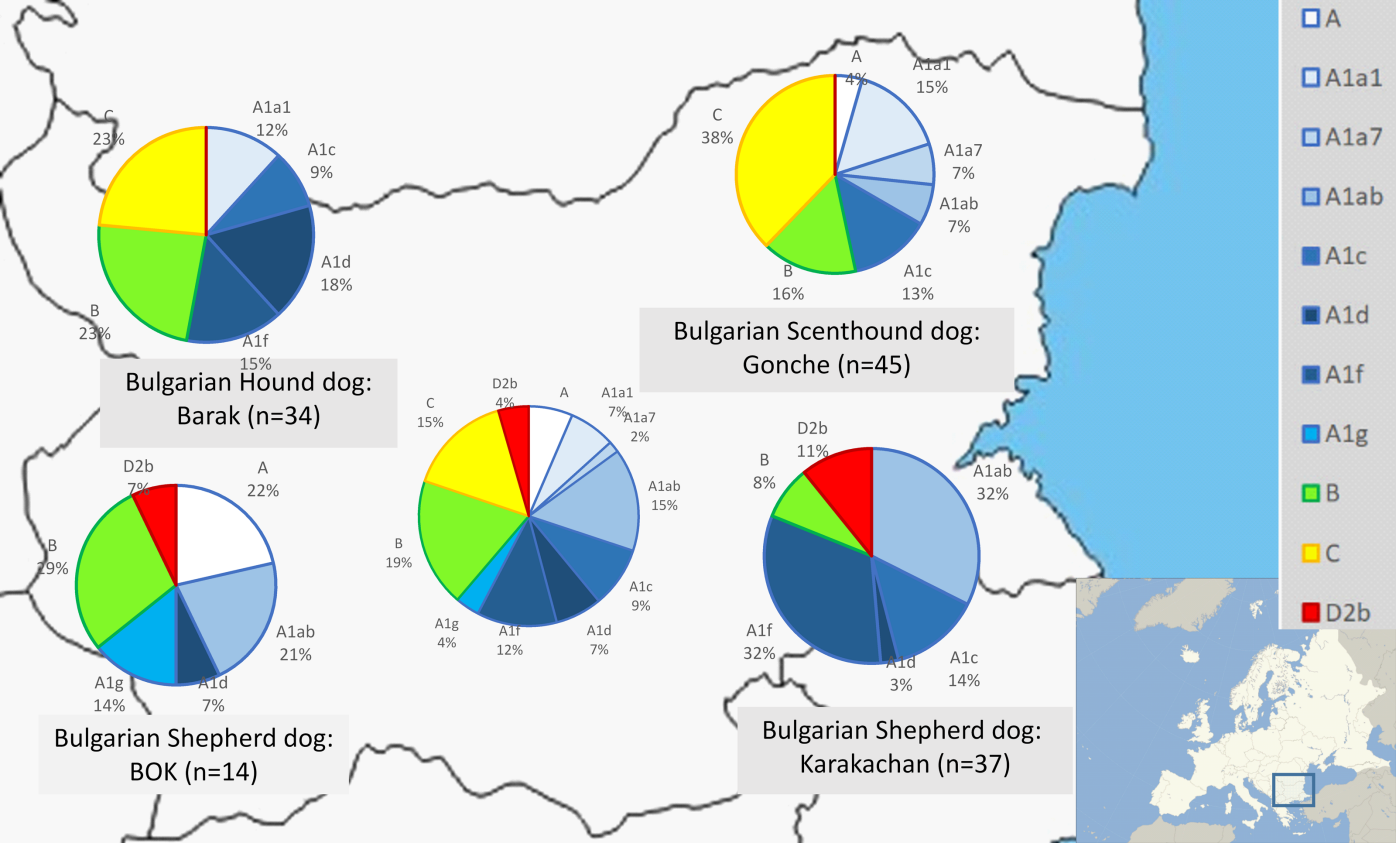
Mitochondrial DNA haplogroup distributions in three native and one modern Bulgarian dog breeds. Classification of the haplogroups are by Duleba et al. (2015) and Song et al. (2016).

We defined each (sub)clade by a specific mutation motif encompassing control region (HVR1). We observed four main clades—A, B, C and D. Within clade A, we identified eight subclades; within clades B and C, we identified a lower diversity compared to clade A, i.e., clade B (B1a1 and B1a4) and clade C (C1b3 and C2). (Fig. 2, Supplemental Information 1).

Subclade A1 was with the highest frequency—54.78%, followed by clades B (18.94%), C (15.33) and D (4.49%) in all dog populations (Supplemental Information 1). In clade D, only subclade D2b was identified with a frequency of 4.49%.

**Figure 3:**
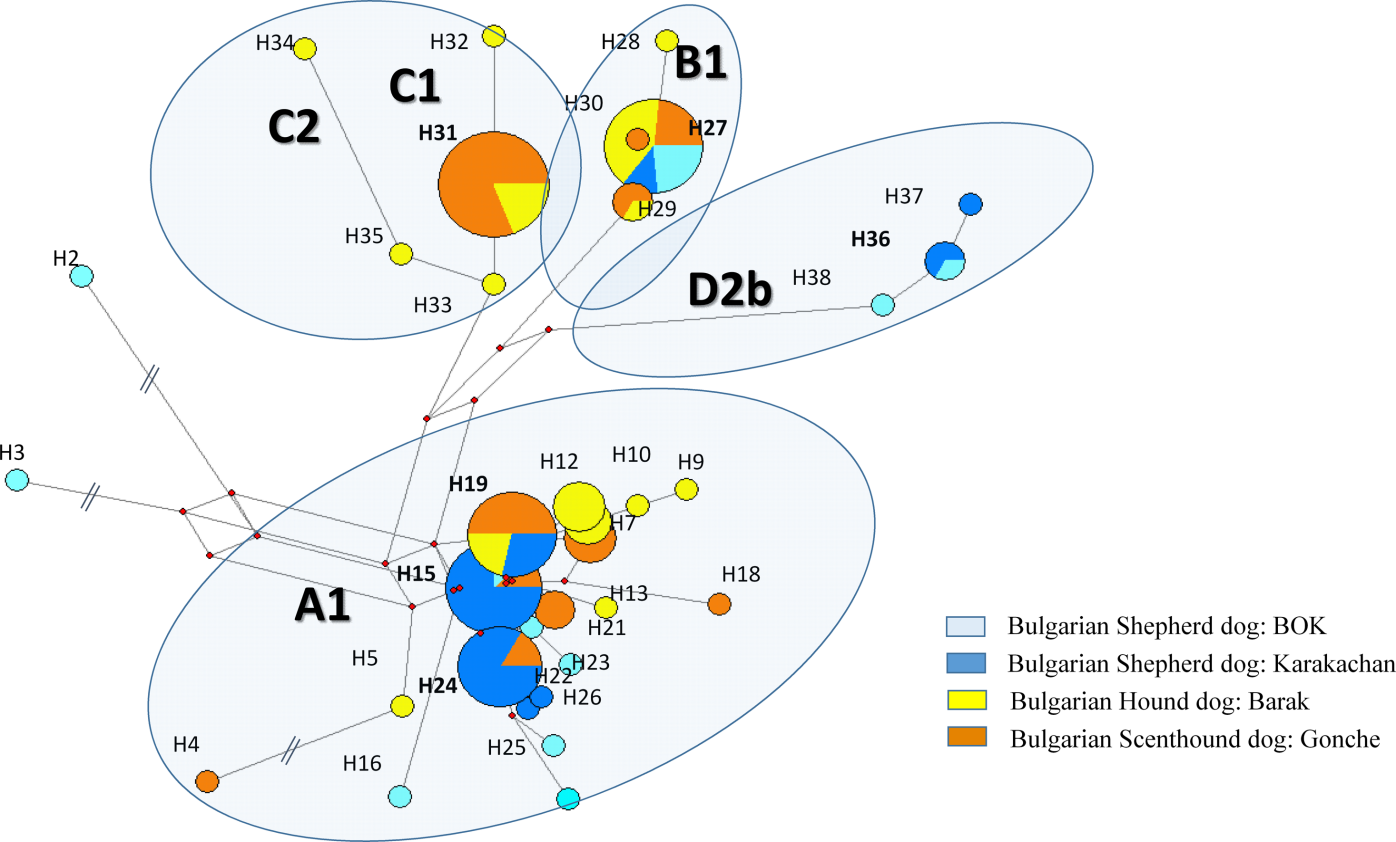
The reduced median network of the main mtDNA haplotypes from three native and one modern Bulgarian dog breeds. The sequence variations and codes of the haplotypes are from Supplemental Information 1.

There were significant differences in the distribution in haplogroups between hunting and shepherd dog breeds (Figs. 2 and 3 and Supplemental Information 1). Clade C was observed in the Bulgarian hunting dog breeds (Bulgarian Scenthound Dog: Gonche and Barak), while subhaplogroup D2b was observed only in shepherd dogs (Bulgarian Shepherd Dog: BOK and Bulgarian Shepherd Dog: Karakachan). Additionally, it was also found that some of the subhaplogroups showed different presence in different dog breeds (Fig. 2).

## Discussion

### Balkan dog breeds—origin and history

Up to date information on the genetic diversity of dogs in South-Eastern Europe is missing. It is not known how much the Balkan dog population is influenced by crossbreeding along migration routes between the Middle East (Anatolia), West Asia and Europe. On the Balkans, native breeds are typical hunting (Bulgarian Barak Hound, Bulgarian Scenthound Dog (Gonche), Hungarian hound (Transylvanian Scenthound), Serbian Hound and Greek Harehound) and shepherd dogs (Bulgarian Shepherd Dog, Greek Sheepdog (Greece), Tornjak (Bosnia and Herzegovina and Croatia), Sharplaninac (FR Macedonia), Akbash Dog (Turkey). All these have a common predecessor from historic or prehistoric time. These breeds diverged are not morphologically distinctive but they are classified separately because of historical reasons related to changing state borders (in the last 200 years). There is no historically described pedigree for these breeds, but it is believed that their predecessor dates back to the times of the Ottoman, the Roman and the Bulgarian Empires, and even to the Hellenic (Greek) and Thracian kingdoms. It should be noted that hunting dogs have been painted during all historic periods as aristocratic dogs, visually similar to scenthound breeds.

### Phylogenetic analysis of the Bulgarian dog population and relationships with surrounding dog populations

Taken together, mtDNA data from the present study showed the presence of all basic European clades in the Bulgarian native dogs. As expected, the majority of samples belong to clade A, especially, European subclades A1 (about 55%) (Figs. 2 and 3). Similarly, there were the typical European subclades B1a1 (about 3%) and B1a4 (about 14%) as well as C1b3 (about 18%) according to the classification system by Duleba et al. (2015). It is not surprising that we found presence of the unique for the Bulgarian Shepherd Dog subclades D2b (about 5%), because subclade D2 is regionally specific for South European dog populations (Duleba et al., 2015; Pires et al., 2006; Pang et al., 2009; Adeola et al., 2017).

We identified 38 haplotypes in all dog breeds. Among them, 19 haplotypes (85%) are identical to the ones available on GeneBank worldwide (Supplemental Information 1) (Cairns et al., 2017; Boyko, Boyko & Boyko, 2009; Duleba et al., 2015; Strakova et al., 2016; Imes et al., 2012; Bekaert et al., 2012; Pires et al., 2006; Verscheure, Backeljau & Desmyter, 2014). Specific for all Bulgarian dog populations is the higher frequency of clade C (about 20%) compared to European dog population (about 10%) (Duleba et al., 2015; Ardalan et al., 2011). Clade C, together with clade D, was predominant in ancient European dogs about 7,000 years ago (63 and 20%, respectively), while most modern European dogs have sequences with haplogroups A and B (64 and 22%, respectively) (Frantz et al., 2016). As a comparison, during the same historical period in South-Western Asia (Anatolia), the frequencies of clades C and D2 were about 7% and 2%, respectively (Ardalan et al., 2011). Otherwise, the subclade D2b from the Bulgarian sets is represented with new specific haplotypes H36 and H37, which differ from other available D2 sequences by parsimony informative substitution T/C at position 15,955 bp, according to ref. sequence accession number NC_002008. Moreover, from all five defined D2 sequences, D2b haplotypes were found in 80% of this set (Supplemental Information 1), which suggests that these haplotypes have been restricted to the Balkans and were isolated a long time ago (probably several millennia ago).

The results, especially the frequency of clade C, may be interpreted as a proof of the conservation of ancient European mitotypes in the studied native Bulgarian dogs.

### Mitochondrial diversity shows dual phylogenetic origin between Bulgarian hunting and shepherd dogs

Regarding the mtDNA analysis of Bulgarian native breeds, we unexpectedly found clear and specific differences between Bulgarian hunting and shepherd dog breeds (Figs. 2 and 3). While clade C was present only in hunting dogs (Bulgarian Hound Dog: Barak and Bulgarian Scenthound Dog: Gonche), the Bulgarian Shepherd Dog was characterized with a Mediterranean specific D2 subclade.

#### Native Bulgarian hunting dogs

The native hunting dogs on the Balkans, as mentioned, are divided into two coat types: rough (long hair) and smooth (short hair). These dogs are known under different breed names and slight morphometric features depending on their distribution on the Balkan countries.

Bulgarian hunting dogs belong to the three main clades A, B and C (Figs. 2 and 3). Both Bulgarian hunting breeds have an unusual high frequency of clade C (Barak—about 24% and Gonche—about 38%). The most prevalent subhaplogroup is C1b3. This suhaplogroup is rarely observed in modern European dog breeds (below 5%) (Fregel et al., 2015). Moreover, clade C is missing in modern scenthounds (greyhounds) (Savolainen et al., 2002). Another characteristic specific only for the Bulgarian hunting dog is the presence of subclades A1a1, A1a1a, A1a1d, A1a1f, A1a1 h A1a7, and B1a1 (Supplemental Information 1). All of these subhaplogroups are observed in European and South-Western Asian dog populations (Fregel et al., 2015). A RM network did not show star-like phylogeny for clades A, B and C (Fig. 2). This means that the origin of these Bulgarian hunting breeds includes at least two separate predecessors—one from ancient East Eurasian dogs carrying clade A or B, and another from West Eurasian carrying clade C, but not the Mediterranean D2 dog subhaplogroups.

#### Native Bulgarian shepherd dogs

There is solid evidence that the Balkan shepherd dogs have directly originated from the now extinct Molossus dog from the Balkan region (Greece, Thrace and Illyric regions) (Kitchell Jr & Kenneth, 2014). Yet one of the basic questions about these guard dogs, regarding their origin, was posed by Aristoteles (Kitchell Jr & Kenneth, 2014) and Xenophon (Cynegeneticus, 2,000 years ago). It has been speculated that these dog breeds may have originated from the Tibetan mastiff, the Anatolian shepherd breeds, the Chinese breeds, etc. Actually, this question still remains open.

In our sample sets we used two shepherd breeds: the native Bulgarian Shepherd Dog Karakachan (BShD) and the newly created Bulgarian Shepherd Dog (BOK), the latter serving as a control. In the last breed typical Central Asiatic dog breeds also contributed genetically.

Our results showed the specific subhaplogroups A1d, A1g and A1f. These subhaplogroups are typical for European dog populations (Fregel et al., 2015). As expected, the mitochondrial DNA profile of the Shepherd Dog (BOK) shared common subhaplogroups with its predecessor—the Karakachan Dog and included some specific Central Asiatic subhaplogroups such as A2a (H1) (Fig. 2). The obtained results clearly show a typical mtDNA profile different from that of the Bulgarian hunting dogs. Subhaplotype D2b is typical for these breeds—about 10% for the Karakachan Dog and about 7% for BOK (Supplemental Information 1). Subclade D2 has also been observed in mountain and shepherd dogs like those in Spain as well as the Estrela Mountain Dog and the Alentejo Shepherd Dog (Portugal), and the Turkish Shepherd Dog: Kangal (Turkey) (Pires et al., 2006; Savolainen et al., 2002; Adeola et al., 2017). All these breeds belong to the European and South-West Asian Molossus group.

## Conclusion

In conclusion, the mtDNA profile of native Bulgarian dogs in the underinvestigated so far South-Eastern Europe has shown typical European haplogroup dissemination. The Bulgarian native dog population is characterized by the highest frequency of clade A (55%), followed by clade B and C (about 18%) and the specific South-European clade D2 (about 10%). Hunting dogs (Gonche and Barak) have a specific mtDNA profile characterized by the presence of clade C and the absence of the Mediterranean subclade D2, as compared to the mountain Shepherd Dog (Karakachan). These data may explain the different phylogenetic origin of two geographically separated European dog populations: Central Europe (hunting dog breeds) and Mediterranean region (mountain shepherd dog).

##  Supplemental Information

10.7717/peerj.5060/supp-1 Supplemental Information 1 Supplementary material

10.7717/peerj.5060/supp-2 Supplemental Information 2 GenBank sequences

## Additional Information and Declarations

### Field Study Permissions

The following information was supplied relating to field study approvals (i.e., approving body and any reference numbers): The experiments were carried out under permissions and the guidelines of the Bulgarian Academy of Sciences and the Bulgarian Ministry of Environment and Waters (no. 627/30.03.2015).

### Data Availability

The following information was supplied regarding data availability: The sequences described here are accessible via GenBank accession numbers MG920357–MG920486. Sequence data can also be found in the Supplemental Information.
